# Liming can decrease legume crop yield and leaf gas exchange by enhancing root to shoot ABA signalling

**DOI:** 10.1093/jxb/erv042

**Published:** 2015-03-04

**Authors:** Shane A. Rothwell, E. David Elphinstone, Ian C. Dodd

**Affiliations:** ^1^The Lancaster Environment Centre, Lancaster University, Lancaster LA1 4YQ, UK; ^2^Myerscough College, Bilsborrow, Preston PR3 0RY, UK

**Keywords:** Abscisic acid, ionome, liming, phosphorus, stomatal conductance, *wilty.*

## Abstract

To meet future requirements for food production, sustainable intensive agricultural systems need to optimize nutrient availability to maximize yield, traditionally achieved by maintaining soil pH within an optimal range (6–6.5) by applying lime (calcium carbonate). However, a field trial that applied recommended liming rates to a sandy loam soil (increasing soil pH from 5.5 to 6.2) decreased pod yield of field bean (*Vicia faba* L. cv. Fuego) by ~30%. Subsequent pot trials, with liming that raised soil pH to 6.3–6.7, reduced stomatal conductance (*g*_s_) by 63, 26, and 59% in *V. faba*, bean (*Phaseolus vulgaris*), and pea (*Pisum sativum*), respectively. Furthermore, liming reduced shoot dry biomass by 16–24% in these species. Ionomic analysis of root xylem sap and leaf tissue revealed a decrease in phosphorus concentration that was correlated with decreased *g*_s_: both reductions were partially reversed by adding superphosphate fertilizer. Further analysis of pea suggests that leaf gas exchange was reduced by a systemic increase (roots, xylem sap, and leaves) in the phytohormone abscisic acid (ABA) in response to lime-induced suboptimal plant phosphorus concentrations. Supplying synthetic ABA via the transpiration stream to detached pea leaves, at the same xylem sap concentrations induced by liming, decreased transpiration. Furthermore, the *g*_s_ of the ABA-deficient mutant pea *wilty* was unresponsive to liming, apparently confirming that ABA mediates some responses to low phosphorus availability caused by liming. This research provides a detailed mechanistic understanding of the physiological processes by which lime application can limit crop yields, and questions the suitability of current liming recommendations.

## Highlight

This paper describes a novel mechanistic understanding of how recommended rates of liming to manage soil pH may cause reduced gas exchange and growth in legumes.

## Introduction

Modern intensive agricultural practices that rely heavily on applying supplementary mineral fertilizers may accelerate naturally occurring soil acidification processes that can impair crop productivity (Bolan *et al.*, 2003; Matsuyama *et al.*, 2005). The principal factors affecting crop productivity in low pH (<5.5) mineral soils are phytotoxicity associated with increased bioavailability of aluminium (Al) and manganese (Mn) (Brady and Weil, 2008), and decreased availability of certain plant nutrients [typically calcium (Ca), magnesium (Mg), potassium (K), and phosphorus (P)]. In acidic mineral soils with a pH <5.5, raising the soil pH to recommended levels (typically pH 6–6.5) would normally be achieved by adding lime (CaCO_3_) (Goulding *et al.*, 2008).

Managing the deleterious effects of soil acidification by liming has long been shown to improve crop yield (Bolton, 1970; Buerkert *et al.*, 1990; Farhoodi and Coventry, 2008). However, liming responses are not always positive, and yield reductions can occur even at recommended application rates. This has been attributed to changes in tissue Ca:Mg ratios (Carran, 1991), Mg fixation within the soil (Sumner *et al.*, 1978), or reduced P availability (Haynes, 1982; Maxwell *et al.*, 2012). More recent work tested the hypothesis that unregulated Ca uptake by the plant increased root xylem sap calcium ion (Ca^2+^) concentration or delivery rate, causing a stomatal limitation of photosynthesis that may limit growth of limed legumes. However, the same concentrations of Ca^2+^ found *in vivo* failed to elicit stomatal closure when fed to detached leaves via the xylem, and it was concluded that an alternative, as yet unidentified, xylem-borne antitranspirant must be regulating stomatal aperture (Rothwell and Dodd, 2014). Preliminary xylem ionomic analysis of these plants suggested that lime application may reduce P uptake, thereby limiting leaf gas exchange.

Phosphorus is an essential plant nutrient that is necessary for many plant processes including synthesis of phospholipids, energy transfer, and enzyme activation (Hawkesford *et al.*, 2012); therefore, inadequate P availability is a major limitation to plant growth and development (Schachtman *et al.*, 1998) and consequently global crop production (Raghothama and Karthikeyan, 2005). It is estimated that 30–40% of global agricultural soils are limited by P availability (Vance *et al.*, 2003) and it is second only to nitrogen (N) in limiting agricultural productivity (Holford, 1997). Although P deficiency might directly limit growth of limed plants, it is not clear how low P availability may limit plant gas exchange.

Phosphorus deprivation decreased stomatal conductance (*g*_s_) of two cultivars of *Capsicum annum* (Davies *et al.*, 1999) and in *Ricinus communis* (Jeschke *et al.*, 1997). However, direct effects of tissue P concentration seem unlikely, as P, N, and S deficiency all elicit stomatal closure, suggesting that these responses are not caused by tissue nutrient levels *per se* (Clarkson *et al.*, 2000) but by a common or centralized response to those deficiencies (Chapin, 1990; Kudoyarova *et al.*, 2015).

It has been suggested that P and other nutrient deficiencies limit both leaf growth and *g*_s_ via decreased tissue water status (Radin and Eidenbock, 1984; Chapin, 1990) caused by reduced root hydraulic conductance (Clarkson *et al.*, 2000). However, maintaining leaf water status by root pressurization did not maintain *g*_s_ or leaf elongation in response to soil drying in *Triticum aestivum* (Gollan *et al.*, 1986), salt stress (100mM NaCl) in *T. aestivum* and *Hordeum vulgare* (Termaat *et al.*, 1985), and in N-deprived *H. vulgare* (Dodd *et al.*, 2002), suggesting that hydraulic signals may not regulate physiological responses. Alternatively, cotton (*Gossypium hirsutum*) plants with a leaf P concentration of 2.3mg g^–1^ dry weight (DW) showed a 2-fold increase in leaf abscisic acid (ABA) concentration in response to soil drying compared with plants with a leaf P concentration of 6.8mg g^–1^ DW (Radin, 1984). Similarly, decreasing the root P concentration from 2.7mg g^–1^ DW to 0.9mg g^–1^ DW (and leaf P concentration from 3.7mg g^–1^ DW to 1.6mg g^–1^ DW) in well-watered *R. communis* plants increased root xylem sap ABA concentration and foliar ABA concentration by 6-fold and 2-fold, respectively (Jeschke *et al.*, 1997). Elevated endogenous levels of ABA, if delivered to the apoplast in the vicinity of the stomatal guard cells, can cause stomatal closure (Hartung *et al.*, 2002), but this hypothesis has never been explicitly tested in P-deprived plants.

Further evidence for the action of phytohormones on a physiological response can be established by seeking to manipulate their endogenous concentrations by using mutants that either are impaired in their ability to synthesize the hormone of interest or are insensitive to its action (Jones *et al.*, 1987; Nagel *et al.*, 1994; Dodd, 2003; Chen *et al.*, 2013). Stomatal conductance of both wild-type and ABA-deficient *wilty* pea was similarly decreased (by 40%) in plants grown at low (0.5mM NO_3_^–^) compared with those grown at high (5mM NO_3_^–^) nitrogen status (Dodd, 2005). Similarly, N-deprived ABA-deficient *flacca* and wild-type tomato had comparably decreased *g*_s_ when compared with N-sufficient plants (Coleman and Schneider, 1996). To the authors’ knowledge, there has been no mutational analysis of the causes of stomatal closure in P-deficient (or limed) plants.

Since previous short-term (4 week) pot trials established that liming an acidic soil to a recommended soil pH decreased shoot biomass (Rothwell and Dodd, 2014), an initial experiment aimed to establish the agronomic implications of this response over the entire crop life cycle. In attempting to understand why crop yield and photosynthesis were decreased in plants grown in limed soil, the tissue and xylem ionic status was investigated. The role of P status in regulating biomass accumulation and leaf gas exchange was investigated by applying factorial combinations of lime and superphosphate. Based on this analysis, it was hypothesized that a liming-induced reduction in P availability decreased gas exchange by increasing root to shoot signalling of the plant hormone ABA. This hypothesis was further tested by measuring transpiration of detached pea leaves that were supplied with the ABA concentrations found endogenously in limed plants, and by measuring *g*_s_ of wild-type (WT) and the ABA-deficient pea (*Pisum sativum*) mutant *wilty* (De Bruijn *et al.*, 1993) grown in limed and unlimed soil.

## Materials and methods

### *Vicia faba* field trial

A field experiment was carried out at Lee Farm, Myerscough College, Lancashire, UK on a site previously established as pasture. The site was chosen as a low pH sandy loam soil (pH 5.5, 46% sand, 32% silt, 9% clay, 13% organic matter) that allowed the application of agronomically significant levels of lime. Treatments were an unlimed control and calcium carbonate- (CaCO_3_) based agricultural lime (J. Arthur Bowers Ltd Coarse Screened Limestone, William Sinclair Horticulture Ltd, Lincoln, UK) that had a neutralizing value (CaO equivalent) of 57%, added at 7 t ha^–1^. The lime application rate was calculated to meet the DEFRA- (2010) recommended target soil pH of 6.5 using the Rothlime online liming calculator (McGrath, 2002). Four plots of each treatment were arranged in a complete randomized design; plot size was 5×3 m with a 2 m buffer zone between plots.

Lime was applied by hand on 7 February 2013 to previously ploughed plots and incorporated into the top 10–15cm of the soil profile using a tractor-mounted rotavator. A crop of *Vicia faba* L. cv. Fuego was drilled 2 months later at a rate of 25–30 seeds m^–2^. Soil samples were taken for pH analysis immediately prior to the lime application and again on 30 May, 13 August, and 21 September 2013. The crop was managed using standard agronomic protection practice which included an application of Bentazon selective herbicide (at recommended rates supplied by the manufacturer) 1 month after drilling to control weeds. At the end of the experiment, pods were collected from two randomly selected 1 m^–2^ quadrats per plot and weighed on a digital hand-held balance to record pod yield.

### Legume pot trials

#### Soil preparation and analysis 

All pot trials used the same 2:1 (v:v) mixture of the low pH sandy loam field soil described above and horticultural grit sand (DA30, Boughton, Kettering, UK), which was used to improve drainage. Field soil–grit sand combinations were homogenized in a cement mixer for 5min, passed through a 10mm sieve, and sterilized (Camplex 68 l, Thermoforce Ltd, Cockermouth, UK) at a minimum temperature of 82 °C to prevent infection from soil-borne pests and diseases. The previously described agricultural lime was applied at a rate of 3g l^–1^ to target a final soil pH of 6.5 as recommended by DEFRA (2010) and converted from t ha^–1^ to g l^–1^ by assuming that soil pH is measured in the top 20cm of the soil profile and 1 ha contains 2 000 000 litres of soil at 20cm depth. Field soil–grit sand and lime combinations were thoroughly homogenized in 15 litre batches for 5min in a cement mixer before incubation in black plastic bags for a minimum of 4 weeks prior to planting to allow the lime reaction to occur.

To prepare soil from both field and pot trials for analysis, samples were homogenized, air-dried, and passed through a 4mm sieve. Soil pH was determined in triplicate using the DEFRA-recommended (MAFF, 1986) method where 20g soil samples were mixed in small plastic containers with 50ml of distilled water, thoroughly stirred, and left for 1h. Soil pH was determined by re-mixing and immediately measuring the suspension with a pH electrode (Orion Sure Flow, Fisher Scientific, Loughborough, UK) and meter (Denver instruments, Bohemia, New York, USA).

#### Plant culture 

In separate experiments, seeds of *V. faba* L. cv. Longpod, *Phaseolus vulgaris* L. cv. Nassau, or *P. sativum* L. cv. Alderman were sown into 1.5 litre (*V. faba*) or 0.8 litre (*P. vulgaris* and *P. sativum*) pots using the control and limed soil described above. In another experiment with *P. sativum* cv. Alderman, one group of control and limed plants received an additional treatment of superphosphate fertilizer (J. Arthur Bowers Ltd) at a rate of 0.59g l^–1^ (equivalent to a 200kg ha^–1^ application rate). In a separate experiment, near-isogenic seeds of the ABA-deficient pea *wilty* mutant (De Bruijn *et al.*, 1993) which show 60-90% lower foliar ABA concentrations than its WT (Dodd et al 2003, Wang et al 1984), its WT, and cv. Alderman were germinated and established as described above.

Plants were initially watered to run-off and weighed after 24h to establish weight at drained capacity, and maintained well-watered by replacing full evapotranspiration (determined gravimetrically) daily and kept in a semi-controlled naturally lit greenhouse with supplementary lighting (supplied by Osram 600W daylight bulbs) for 12h and 22 °C/16 °C minimum day/night temperature at the Lancaster Environment Centre.

#### Physiological measurements 

Stomatal conductance was recorded 24h prior to harvest on the third or fourth leaf pair numbered from the base (*V. faba* and *P. sativum*), or the first tri-foliate leaf (*P. vulgaris*) using an AP4 diffusive porometer (Delta-T Devices, Cambridge, UK). Two readings were taken per plant and averaged. Measurements were made between 11:00h and 13:00h on the abaxial leaf surface.

In the superphosphate addition experiment, gas exchange [*g*_s_ and photosynthesis (Pn)] was recorded using infra-red gas analysis (6400Xt Li-Cor Portable Photosynthesis System, Lincoln, NE, USA) on one leaflet of leaf pair four. Instrument settings were ambient CO_2_ levels (390 μl l^–1^), 600 μmol m^–2^ s^–1^ phosynthetic photon flux density (PPFD), a cuvette temperature of 22 °C, and ambient humidity.

In the *P. sativum* superphosphate experiment, leaf water potential (Ѱ_leaf_) of one leaf of leaflet pair four was measured by thermocouple psychrometry. Leaf discs of 8mm diameter were punched from the mid-lamina, placed immediately on clean sample holders, and then wrapped in aluminium foil to minimize water loss. When all samples had been collected, they were unwrapped and loaded into C52 sample chambers (Wescor Inc., Logan, UT, USA), incubated for ~3h, then voltages were read with a microvolt meter (model HR-33T; Wescor Inc.). Voltages were converted into water potentials based on calibration with salt solutions of known osmotic potential.

Root (*P. vulgaris*) or root and leaf (*P. sativum*) xylem sap samples were collected for ionomic and/or hormonal analysis at flow rates closely matching *in vivo* transpiration rates, determined gravimetrically 1–2h prior to sampling, using techniques described in more detail previously (Rothwell and Dodd, 2014). Briefly, de-topped 3-week-old *P. vulgaris* plants were placed in a Scholander pressure chamber and sap collected using an appropriate over-pressure (0.2–0.5MPa). For *P. sativum*, leaf xylem sap was collected from a small V-shaped section cut from the mid-rib of one leaflet of leaflet pair four, using plants grown in a whole-plant pressure chamber. Root xylem sap was collected after subsequent excision of the shoot 3cm above the soil surface, at sequentially increasing 0.1MPa pressures until the appropriate sap flow rate was achieved. Sap samples were immediately frozen in liquid nitrogen and stored at –80 °C prior to analysis.

Plants in all experiments were harvested at 3–4 weeks old. Roots were collected, washed clear of soil, and checked for nodulation, though this was not observed. Both shoot and root material was then dried at 80 °C for 1 week to record dry weight and stored in air-tight containers to provide samples for nutrient and hormone analysis.

#### Plant analyses 

For tissue nutrient analysis, all leaves or roots present at the time of harvest were collected to provide enough sample material, oven dried at 80 °C for 7 d, and ground to a fine powder using a ball mill (Retsch MM400, Retsch UK Limited, Castleford, West Yorkshire, UK). Samples were then subjected to microwave-assisted acid digestion (Mars-5 Xpress microwave-accelerated reaction system, CEM Corporation, Matthews, NC, USA) in trace metal grade HNO_3_ (Sigma-Aldrich, Dorset, UK) for 30min at a maximum temperature of 200 °C. To prepare samples for analysis, the digestate was diluted to a final 2% (v/v) HNO_3_ concentration with Millipore water and filtered through a 0.45 μm syringe filter. Xylem sap samples were diluted directly in a 2% (v/v) HNO_3_ solution, and filtered prior to analysis. Macronutrients (Ca, K, Mg, P, and S) were analysed using inductively coupled plasma-optical emission spectrometry (ICP-OES; iCAP 6300, Thermo Scientific, MA, USA) and compared against standards of a known range of concentrations, and corrected, if required, using determinations from blank samples run in the microwave digestion.

Leaf and root xylem sap and tissue ABA concentrations were determined by competitive radioimmunoassay (RIA) as previously described by Quarrie *et al.* (1988) using radiolabelled ABA (dl-*cis/trans* [^3^H]ABA) and the antibody MAC 252 (Dr Geoff Butcher, Babraham Institute) that has high specificity for the free acid of (+)-2-*cis*-ABA (Barrieu and Simonneau, 2000). To prepare plant tissue for analysis, samples were freeze-dried and ground to a fine powder using dissecting scissors. Both leaf and root tissue samples were extracted at a ratio of 1:25 in distilled water by shaking overnight at 4 °C on a mechanical shaker. Leaf and root xylem sap samples were analysed as collected. A spike dilution test of both xylem sap and aqueous extracts of *P. sativum* tissue (Bacon, 2001) indicated the absence of immunoreactive contamination.

#### Detached leaf transpiration bioassays 

Eight uniformly germinated seeds of *P. sativum* L. cv. Alderman were established in 5 litre pots using the unlimed control 2:1 field soil:grit sand combination described above. When established (2–3 weeks), fully expanded leaflet pairs including petioles were detached 4–5h into the photoperiod using a razor blade and immediately re-cut under distilled water to prevent embolism. Maintaining a meniscus of water on the cut petiole surface, the leaflets were then placed in a 1.5ml Eppendorf tube containing an artificial xylem sap solution containing: 3mM KNO_3,_ 1mM KH_2_PO_4_, 1mM K_2_HPO_4_, 1mM CaCl_2_, 0.1mM MnSO_4_, and 0.1mM MgSO_4_ (as in Dodd *et al.*, 2003). A transpiration dose response to ABA was established by adding ABA at concentrations of 0, 10, 50, and 100nM to the artificial xylem sap. The *P. sativum* leaflets (placed in small glass vials to allow them to sit upright) were randomly placed in a controlled environment growth chamber with fan-assisted air flow at a temperature of 24 °C with a relative humidity of ~60%. Vials were weighed on a four-point analytical balance every 50min over a 5h period to determine transpiration rates gravimetrically. At the end of the assay, leaflet area was recorded using a leaf area meter (Li-3050A, Li-Cor, Lincoln, NE, USA) to normalize transpiration rates.

### Statistical analysis

A Student *t*-test was used to determine significant effects of lime on soil pH and pod yield; soil pH, biomass accumulation, and *g*_s_; and ionomic composition of leaf tissue and xylem sap. Two-way analysis of variance (ANOVA) determined significant treatment effects of lime and superphosphate fertilizer and effects of lime and genotype. Regression analysis determined correlations, and linear, second-order polynomial or hyperbolic decay regression lines were fitted as appropriate where significant. All analyses used Minitab v16 software.

## Results

### *Vicia faba* field trial

**Fig. 1.**
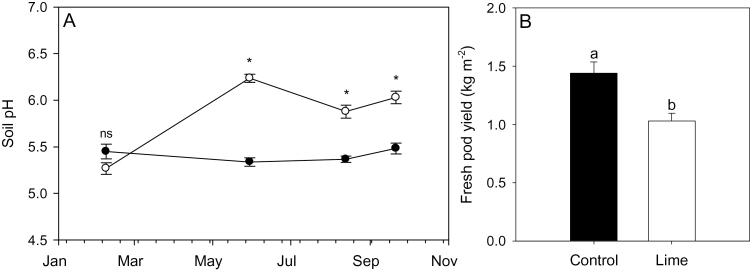
Soil pH of limed (open circles) and unlimed controls (filled circles) during the *Vicia faba* cropping cycle (A). Total fresh pod yield of limed and unlimed (control) *V. faba* recorded 21 weeks after drilling in the field trial (B). Data are means ±SE of four treatment plots. Asterisks (A) and different letters above bars (B) indicate a significant difference (*P*<0.05) as determined by a Student *t*-test.

Soil samples taken 16 weeks after agricultural lime application showed that soil pH increased from ~5.5 to 6.2 (Fig. 1A), slightly less than the pH 6.5 target. Later in the growing season (26 and 32 weeks after application), the pH had dropped slightly in the limed treatment to values between 5.8 and 6. Throughout crop growth, soil pH was significantly higher in the limed treatment. At harvest, liming had reduced fresh pod yield of *V. faba* by 28% (Fig. 1B) from 1.44kg m^–2^ to 1.03kg m^–2^ (21 weeks after drilling) when compared with unlimed controls. Although liming increased soil pH to levels that were considered optimal for growth throughout the entire growing season, pod yield was significantly decreased.

### Pot trials

Liming significantly reduced shoot biomass of *V. faba*, *P. vulgaris*, and *P. sativum* in pot trials by 24, 22, and 16%, respectively, and reduced *g*_s_ by 63, 26, and 59%, respectively (Table 1). Ionomic analysis of leaf tissue (*V. faba*) or root xylem sap (*P. vulgaris* and *P. sativum*) revealed a consistent halving of P concentrations in limed plants (Table 2). Liming significantly increased Ca concentration only in *P. vulgaris* root xylem sap, though none of the other macronutrients measured (K, Mg and S) changed in response to liming. Thus liming consistently decreased biomass accumulation and *g*_s_ of several legume species, but, of the macronutrients analysed, only P status was consistently decreased.

**Table 1.** *Soil pH, shoot dry biomass, and stomatal conductance of limed and unlimed (control*) Vicia faba, Phaseolus vulgaris*, and* Pisum sativum *in pot trials* Data are means ±SE of three replicates for soil pH and 11–12 (*V. faba* and *P. vulgaris*) or 5–6 (*P. sativum*) replicates for biomass and stomatal conductance.

Species	Soil pH		Shoot dry biomass (g)			Stomatal conductance (mmol m^–2^ s^–1^)		
	Control	Lime	Control	Lime	% change	Control	Lime	% change
*V. faba*	5.75±0.03 a	6.29±0.01 b	1.98±0.10 a	1.50±0.16 b	–24	287±35 a	106±18 b	–63
*P. vulgaris*	5.78±0.01 a	6.37±0.02 b	1.96±0.14 a	1.53±0.11 b	–22	626±29 a	462±22 b	–26
*P. sativum*	6.00±0.04 a	6.67±0.02 b	0.89±0.04 a	0.75±0.01 b	–16	303±34 a	125±10 b	–59

Different letters indicate significant differences (*P*<0.05) within each species between treatments as determined by a Student *t*-test.

**Table 2.** *Ionomic analysis of limed and unlimed (control) leaf tissue (mg g* ^*–1*^ *DW;* Vicia faba*) or root xylem sap (mM;* Phaseolus vulgaris *and* Pisum sativum*) in pot trials* Data are means ±SE of five (*V. faba*), 10 (*P. vulgaris*), or three (*P. sativum*) replicates

Species	K		Ca		Mg		S		P	
	Control	Lime	Control	Lime	Control	Lime	Control	Lime	Control	Lime
*V. faba*	24.9±1.86	25.5±2.02	8.64±0.95	10.3±0.77	2.45±0.23	2.66±0.14	NA	NA	3.24±0.18	1.63±0.08*
*P. vulgaris*	5.46±0.32	6.06±0.38	1.19±0.06	1.91±0.09*	0.59±0.02	0.52±0.03	0.19±0.02	0.15±0.01	0.54±0.04	0.23±0.01*
*P. sativum*	8.71±1.33	9.76±0.94	1.00±0.06	0.92±0.01	0.40±0.04	0.29±0.02	0.40±0.02	0.35±0.03	0.28±0.03	0.16±0.01*

NA denotes that the sample was not analysed and * indicates where lime had a significant (*P*<0.05) effect on nutrient concentration.

**Fig. 2.**
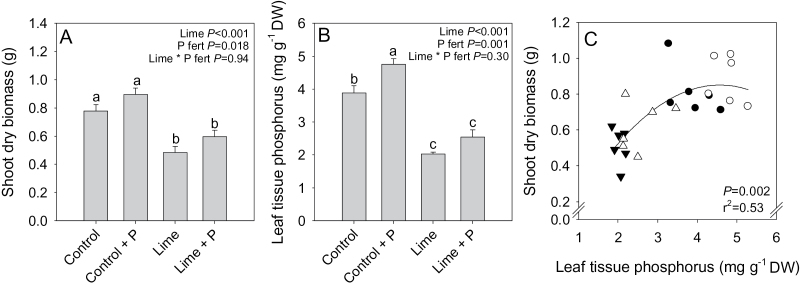
Shoot dry biomass (A) and leaf tissue phosphorus concentration (B) of control ●, control+P fertilizer ○-, lime ▼-, or lime+P fertilizer △-treated *Pisum sativum*, and relationship between shoot dry biomass and leaf tissue phosphorus concentration (C). Data are means ±SE of 8–10 (A) or six (B) replicates. Two-way ANOVA results (*P*-values reported) are indicated in (A) and (B), with different letters above bars indicating significant differences as determined by Tukey pair-wise analysis. Data points in (C) represent individual plants with a second-order polynomial regression line fitted, with *P*-values and *r* ^2^ shown.

Lime and superphosphate fertilizer both had significant, independent effects on both shoot biomass and tissue P concentration. Again, liming significantly reduced shoot dry biomass of *P. sativum* (Fig. 2A) by 38%, but applying superphosphate fertilizer increased shoot dry biomass (by 15–23%). Similarly, liming approximately halved leaf tissue P concentration when compared with unlimed controls (Fig. 2B), but applying superphosphate fertilizer increased leaf P concentration (by ~25% in both control and limed soil). Thus shoot dry biomass significantly increased with leaf tissue P concentration (Fig. 2C).

**Fig. 3.**
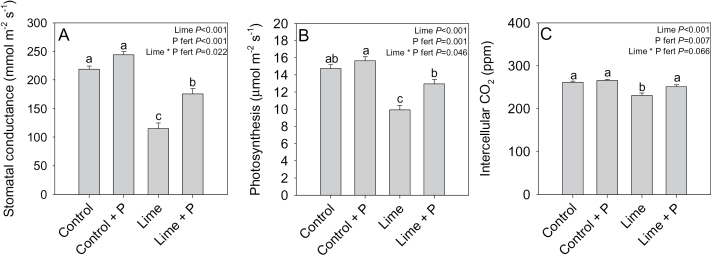
Stomatal conductance (A), net photosynthesis (B), and intercellular CO_2_ concentration (C) of control, control+P fertilizer-, lime-, or lime+P fertilizer-treated *Pisum sativum*. Data are means ±SE of 8–10 replicates with two-way ANOVA results (*P*-values reported) shown. Different letters above each bar indicate significant differences (*P*<0.05) as determined by Tukey pair-wise analysis.

In *P. sativum*, the liming treatment approximately halved *g*_s_ when compared with the unlimed controls (Fig. 3A). Applying superphosphate fertilizer partially restored *g*_s_ in limed plants, but had no significant effect on controls, as signified by the lime×P fertilizer interaction (*P*=0.02). Similarly, liming inhibited net photosynthesis by 32% compared with unlimed controls, but superphosphate fertilizer restored Pn in the limed plants to near control levels (Fig. 3B). Again, a significant lime×P fertilizer interaction (*P*=0.046) indicated that superphosphate fertilizer only enhanced Pn in the limed plants and not in the control group. Limed plants also had a 12% lower leaf intercellular CO_2_ concentration (Fig. 3C) when compared with controls, which was restored to control levels by applying superphosphate fertilizer. Thus liming limited photosynthesis by decreasing *g*_s_ and leaf intercellular CO_2_ concentration, but this could be reversed by superphosphate fertilizer application.

**Fig. 4.**
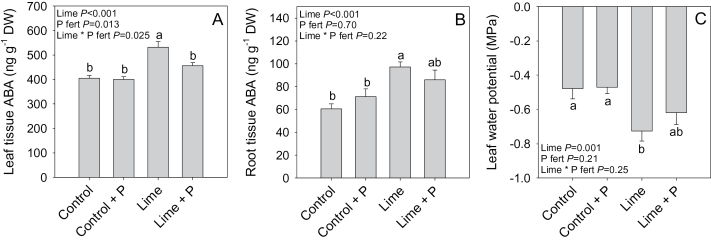
Leaf (A) and root (B) tissue ABA concentrations and leaf water potential (C) of control, control+P fertilizer-, lime-, or lime+P fertilizer-treated *Pisum sativum*. Data are means ±SE of 8–10 replicates with two-way ANOVA results (*P*-values reported) shown. Different letters above each bar indicate significant differences (*P*<0.05) as determined by Tukey pair-wise analysis.

In *P. sativum*, liming increased leaf and root tissue ABA concentration by 31% and 62%, respectively (Fig. 4A, B). Applying superphosphate fertilizer restored leaf tissue ABA levels to control values in the limed treatment, but had no significant effect on plants grown in unlimed soil, as indicated by a significant lime×P fertilizer interaction (*P*=0.025). Applying superphosphate fertilizer had no effect on root ABA concentration in the control treatment although it did partially reduce ABA concentrations of limed plants by ~12%. Liming also reduced Ѱ_leaf_ by 0.25MPa (Fig. 4C) and superphosphate fertilizer partially restored Ѱ_leaf_ to control levels by 0.12MPa, but had no effect on plants grown in unlimed soil (Fig. 4C). Thus liming increased tissue ABA concentration and lowered Ѱ_leaf_, but these effects could be fully or partially reversed by superphosphate fertilizer application.

**Fig. 5.**
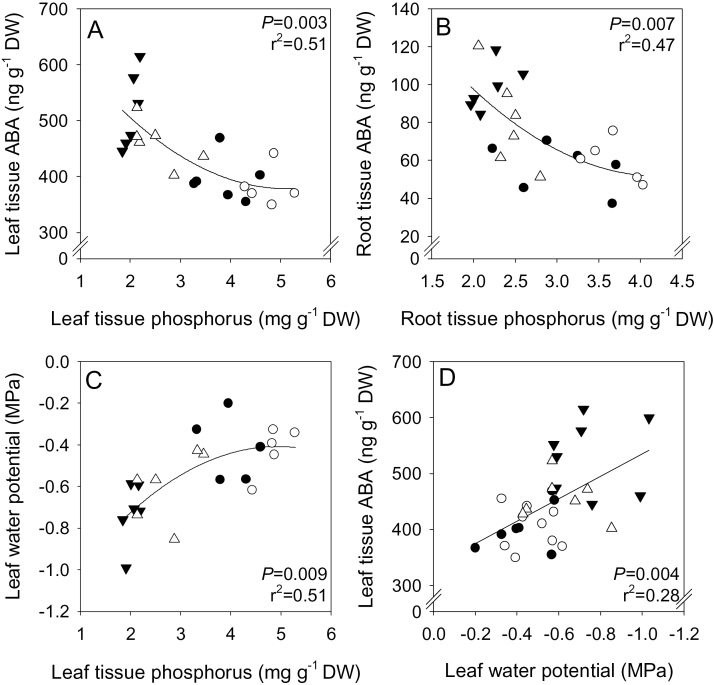
Relationship between leaf (A) and root (B) tissue ABA concentration and phosphorus concentration, leaf water potential and leaf tissue phosphorus concentration (C), and leaf water potential and leaf ABA concentration (D) in limed (filled triangles), limed + P fertilizer (open triangles), unlimed (filled circles), and unlimed + P fertilizer (open circles) *Pisum sativum*. Data points represent individual plants, with second-order polynomial (A, B, C) and linear (D) regression lines fitted, with *P*-values and *r* ^2^ reported.

**Fig. 6.**
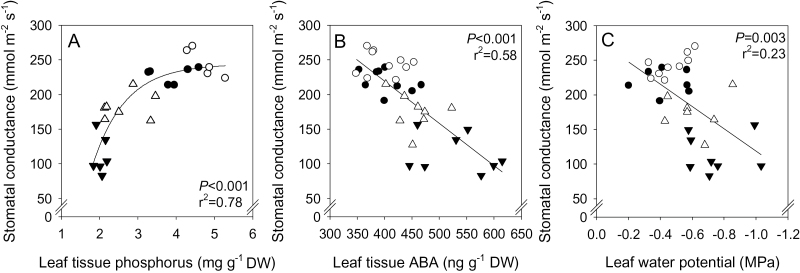
Relationship between stomatal conductance and leaf tissue phosphorus concentration (A), leaf water potential (B), and leaf tissue ABA concentration (C) of lime- (filled triangle), lime+P fertilizer- (open triangle), control (filled circle), and control+P fertilizer- (open circle) treated *Pisum sativum*. Data points represent individual plants with hyperbolic decay (A) or linear (B, C) regression lines fitted, and *P* values and *r* ^2^ reported.

Leaf tissue ABA was correlated (*P*=0.003; *r* ^2^=0.51) with foliar P concentration (Fig. 5A), and root tissue ABA (*P*=0.007; *r* ^2^=0.47) with root tissue P concentration (Fig 5B). Ѱ_leaf_ was also correlated (*P*=0.009; *r* ^2^=0.51) with leaf tissue P concentration (Fig. 5C) and, although data were more scattered, Ѱ_leaf_ was negatively correlated (*P*=0.004; *r* ^2^=0.28) with foliar ABA concentrations (Fig. 5D). Across all treatments, stomatal closure was highly correlated with both decreased foliar P (*P*<0.001; *r* ^2^=0.78) and increased ABA (*P*<0.001; *r* ^2^=0.58) concentrations (Fig. 6A, B), and Ѱ_leaf_ was weakly correlated (*P*=0.004; *r* ^2^=0.22) with *g*_s_ (Fig. 6C). Given the multiplicity of significant correlations, resolving the physiological significance of increased ABA status required additional experiments focusing on the relationship between *g*_s_ and ABA.

**Fig. 7.**
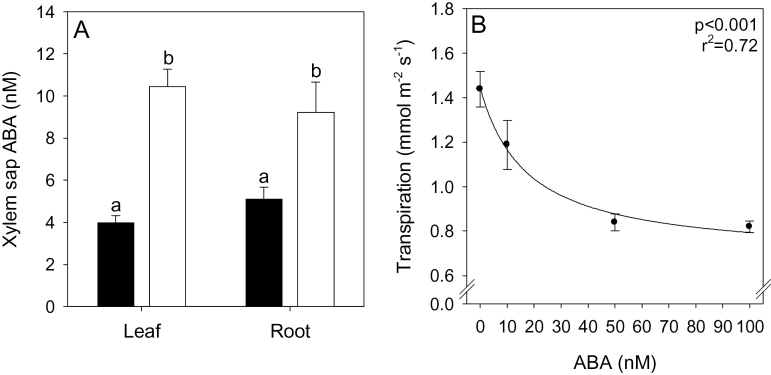
Root and leaf xylem sap ABA concentration (A) of limed (open bars) or unlimed (filled bars) *Pisum sativum* grown in a whole-plant pressure chamber. (B) The relationship between artificial xylem sap ABA concentration and transpiration rate in detached leaflets of *P. sativum*. Data are means ±SE of 5–6 (A) and 6–8 (B) replicates; different letters in (A) indicate significant differences (*P*<0.05) as determined by a Student *t*-test. A hyperbolic decay regression line is fitted in (B), with *P*-values and *r* ^2^ reported in the top right of the panel.

To determine whether root to shoot ABA signalling was also affected by liming, xylem sap was collected from the roots and leaves of *P. sativum* plants grown in specialized pressure pots. Both leaf and root xylem sap ABA concentrations were approximately doubled from ~ 4–5nM to ~10nM (Fig. 7A). Supplying 10nM ABA to detached *P. sativum* leaves via the transpiration stream decreased the leaf transpiration rate by 17% compared with that of leaves supplied with artificial xylem sap alone (Fig. 7B), indicating that this ABA concentration is physiologically active.

**Fig. 8.**
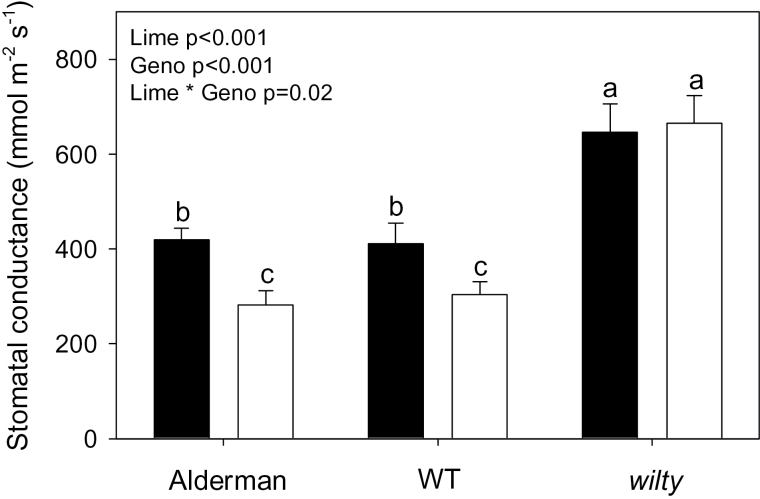
Stomatal conductance of limed (open bars) or unlimed (filled bars) *Pisum sativum* cv. Alderman (Ald), ‘wild-type’ (WT) or *wilty*. Data are means ±SE of 7–8 replicates, and two-way ANOVA results (*P*-values reported) are shown. Different letters above each bar indicate significant differences (*P*<0.05) as determined by Tukey pair-wise analysis.

Further evidence that ABA was involved in decreasing *g*_s_ of limed plants was sought by comparing stomatal responses of wild-type and ABA-deficient *wilty* peas. Liming decreased *g*_s_ of both a commercial cultivar (Alderman) and the WT by ~25%, but had no significant effect on *g*_s_ of the *wilty* pea, as confirmed by a significant genotype×lime interaction (*P*=0.02; Fig. 8.). Thus ABA deficiency prevented the normal stomatal responses of limed plants.

## Discussion

Conventional wisdom suggests that soil pH for arable crops should be maintained between 6 and 6.5 to maximize nutrient availability (Brady and Weil, 2008; Goulding *et al.*, 2008; DEFRA, 2010) and crop yields, and that lime should be applied to correct for excessive soil acidity (pH <5.5). Accordingly, lime was applied in the field trial, which maintained a mean soil pH value of ~6.2 throughout the cropping cycle (Fig. 1A). This pH would be expected to improve crop yield (Bolan *et al.*, 2003) and should have avoided the yield penalties characteristic of overliming (usually generating soil pH values >8) typically caused by unavailability of iron (Mengel, 1994), P and Mn (Kerley, 2000), and boron (Bartlett and Picarell, 1973). Paradoxically, lime application decreased *V. faba* fresh pod yield (Fig. 1B). This was unlikely to be a unique response of this species, since pot trials in the same soil with several legume species (*V. faba*, *P. vulgaris*, and *P. sativum*; Table 1) all showed decreased shoot biomass in response to lime application. While further studies are needed to determine how common this response may be in a range of soil types, this investigation focused on determining the physiological reasons for this lime-induced yield suppression.

Decreased yield following liming has been attributed to fixation of available Mg (Sumner *et al.*, 1978 Myers *et al.*, 1988) or tissue Ca:Mg ratios (Carran, 1991), yet plant uptake of these elements (as assayed by tissue and xylem ionomic analysis) was similar in limed and control plants (Table 2). Previous studies with this soil excluded a role for excessive Ca uptake [Ca is a potent antitranspirant (De Silva *et al.*, 1985)] in limiting leaf gas exchange and biomass accumulation of legume crops (Rothwell and Dodd, 2014). However, tissue or xylem sap P concentrations of limed plants were consistently decreased (Table 2), as in previous observations where liming decreased yield (Haynes, 1982; Maxwell *et al.*, 2012). While further investigations of soil P dynamics seem advisable, adding CaCO_3_ could precipitate large quantities of CaHPO_4_, thereby making P unavailable to the plant (Delgado and Torrent, 2000). The importance of P in regulating plant responses to liming was established by supplying excessive quantities (200kg ha^–1^ P) of P fertilizer to limed plants, which partially reversed the negative effects of lime on *g*_s_ (Fig. 3A), if not biomass (Fig. 2A). Thus it seemed essential to establish the physiological mechanisms by which lime-induced suboptimal P concentrations may decrease biomass accumulation (Fig. 2C) and gas exchange (Table 1; Fig. 3).

While decreased P status may directly regulate biomass accumulation, since considerable quantities of P are needed for nucleic acid formation and phospholipid synthesis (Veneklaas *et al.*, 2012), it seems less likely that P *per se* directly regulates *g*_s_. Certainly, P-deprived plants showed reduced *g*_s_ and transpiration (Jeschke *et al.*, 1997; Davies *et al.*, 1999), and *g*_s_ declined with leaf tissue P concentration (Fig. 6A), suggesting that P concentration *per se* may directly cause or act as a signal for stomatal closure. Although changes in xylem ionomic composition might act as root to shoot signals that influence stomatal aperture (Bahrun *et al.*, 2002), responses of xylem phosphate concentration to soil drying seem rather variable, with increased and decreased concentrations reported (Perez-Alfocea *et al.*, 2011). Similarly, xylem concentrations of a range of measured cations and anions (including phosphate) were not correlated with *g*_s_ at the onset of soil drying (Gollan *et al.*, 1992), suggesting that changes in xylem sap ion concentration are unlikely to regulate stomatal responses directly (Schurr *et al.*, 1992). Alternatively, foliar P concentration might affect stomata by decreasing the CO_2_ fixation rate by impairing photochemical efficiency. However, when P is withheld from the plant, Ci values typically increase (e.g. Jacob and Lawlor, 1991, 1993). In the current experiment, Ci values of limed plants actually decreased (Fig. 3C), suggesting that decreased net photosynthesis results from a reduced CO_2_ flux into the substomatal cavity caused by stomatal closure (Chaves *et al.*, 2002).

Common stomatal responses to multiple individual nutrient deficiencies (N, P, and K) in well-watered plants (Clarkson *et al.*, 2000) make it more plausible that any signal affecting stomatal behaviour acts as part of a centralized response (Chapin, 1990). Hence, the significant negative correlation between tissue P concentrations and *g*_s_ (Fig. 6A) may simply reflect plant P status and may not be causative. Previous work that detected lime-induced stomatal closure argued that an unidentified xylem-borne signal was responsible (Rothwell and Dodd, 2014).

Decreased leaf water status under suboptimal P levels mediated by reduced root hydraulic conductance (Radin and Eidenbock, 1984) may limit *g*_s_ (Clarkson *et al.*, 2000). Although liming decreased both Ѱ_leaf_ and *g*_s_ (Fig. 6C), the *g*_s_ of *P. sativum* is not always closely linked to leaf water status. Flooding decreased *g*_s_ without changing leaf water status (Zhang and Zhang, 1994), and soil drying increased Ѱ_leaf_ by ~0.2MPa, which was thought to be caused by stomatal closure (Belimov *et al.*, 2009). Furthermore, *g*_s_ of low-N- (1mM) supplied tomato (*Solanum lycopersicum*) was 27% lower than that of N-sufficient (10mM) plants without any change in Ѱ_leaf_ (Guidi *et al.*, 1998). Thus it seems unlikely that reduced Ѱ_leaf_ of *P. sativum* caused stomatal closure of limed plants, and instead enhanced concentrations of the phytohormone ABA, a signal common to many nutrient stresses (Vysotskaya *et al.*, 2008), may be important.

Liming increased ABA concentrations in both root and leaf tissues (Fig. 4) and root- and leaf-derived xylem sap (Fig. 7) of *P. sativum*, apparently in response to decreased P availability (Fig. 5A, B). Furthermore, additional P fertilizer prevented foliar ABA accumulation in limed plants (Fig. 4A). In *P. sativum*, foliar ABA concentrations increased below a threshold leaf tissue P concentration of ~2.5mg g^–1^ DW (Fig. 5A). This value appears to vary with species, since P-deprived *R. communis* (P concentration of 1.6mg g^–1^ DW) had a 2-fold increase in leaf ABA concentration (Jeschke *et al.*, 1997), while P-deprived *G. hirsutum* (P concentration of 2.3mg g^–1^ DW) did not show elevated ABA concentrations compared with control plants containing 6.8mg g^–1^ DW (Radin, 1984). This suggests that it is not tissue P concentration *per se*, but a secondary signal that triggers foliar ABA accumulation.

Reduced Ѱ_leaf_ under suboptimal P conditions may trigger leaf ABA synthesis (Zeevaart and Creelman, 1988). Although leaf tissue ABA and Ѱ_leaf_ were weakly correlated (Fig. 5D), significant leaf synthesis of ABA is not thought to occur until leaf water status reaches zero turgor (Pierce and Raschke, 1981); therefore, foliar water deficit is unlikely to be the principal signal triggering foliar ABA accumulation. An alternative argument is that increased root ABA accumulation in response to liming (Figs 4B, 5B) and its export in xylem sap (Fig. 7A) probably influences stomatal behaviour (Hartung *et al.*, 2002).

Unequivocally determining the site of increased ABA production in the current experiments is difficult. Reciprocal grafting experiments of WT plants with ABA-deficient mutants (Albacete *et al.*, 2015) including those with the ABA-deficient *wilty* pea (Wang *et al.*, 1984) have generally established that an ABA-deficient root system has little impact on xylem ABA concentration or stomatal closure in response to soil drying. However, hormone flow modelling techniques (*sensu* Jeschke and Pate, 1991) indicate that in well-watered P-deprived plants, root biosynthesis contributes 82% of xylem exported ABA as opposed to being a moderate sink for ABA metabolism under P-sufficient conditions (Jeschke *et al.*, 1997). Since liming increased both root and leaf xylem sap ABA concentrations equally over unlimed controls (Fig. 7A), and because significant quantities of leaf-synthesized ABA are unlikely to be recycled via the phloem under P deficiency (Jeschke *et al.*, 1997), it is most likely that increased ABA under lime-induced suboptimal P is root synthesized.

Irrespective of the source of this additional ABA in the transpiration stream, the concentrations detected (10nM) in both root and leaf xylem sap of limed *P.* *sativum* plants (Fig. 7A) were sufficient to decrease the transpiration rate of detached leaves (Fig. 7B), consistent with previous detached leaf experiments in pea (Dodd *et al.*, 2008). Further evidence that the decrease in *g*_s_ of limed plants was ABA mediated was provided by observations that *g*_s_ of the ABA-deficient *wilty* was not affected by liming, whereas its WT and a commercial cultivar showed partial stomatal closure following liming (Fig. 8), apparently confirming that ABA induces stomatal closure.

**Fig. 9.**
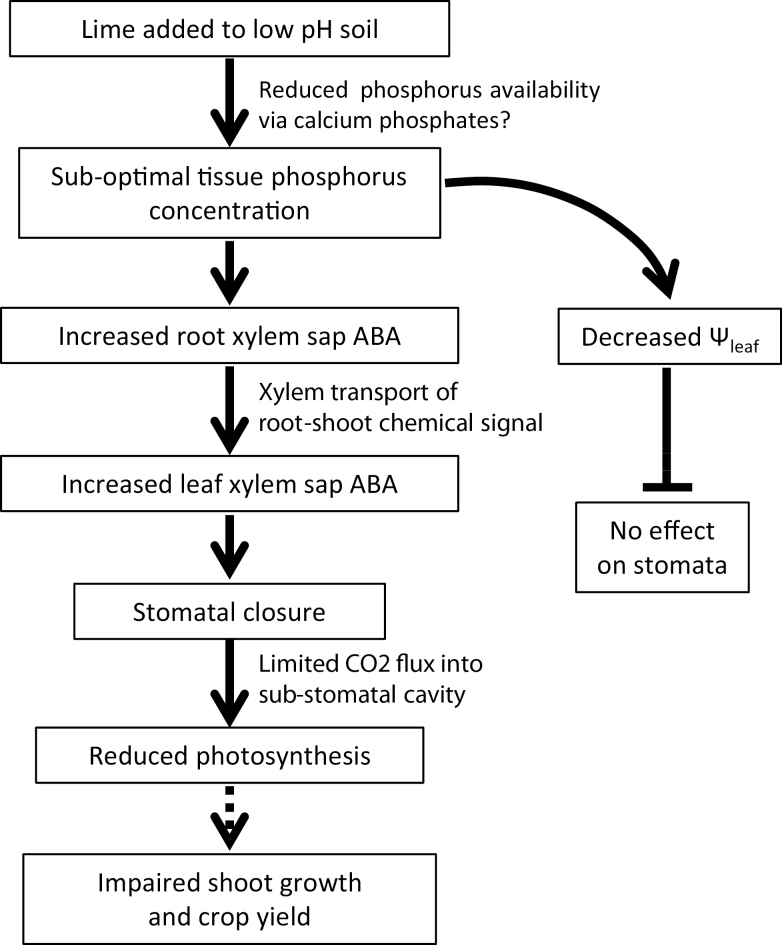
Conceptual model detailing the mechanistic understanding of the physiological processes by which lime can limit gas exchange and shoot growth. Solid lines represent processes established in this study and dashed lines indicate probable mechanisms.

Substantial evidence is required to establish convincingly the physiological significance of any plant hormone in a given response, according to Jackson (1993). In fulfilling these criteria, this study correlates stomatal closure and foliar ABA concentration *in vivo* (Fig. 6B) and duplicates this response in an isolated system (Fig. 7B). Moreover, partially excluding ABA from limed *P. sativum* plants via the ABA-deficient *wilty* pea prevented the typical stomatal response to limed soil (Fig. 8). Taken together, this provides strong evidence that reduced gas exchange in legumes in response to lime-induced suboptimal P concentrations is mediated by the plant hormone ABA, and that this response limits photosynthesis, biomass accumulation, and crop yields (Fig. 9). Re-evaluating liming recommendations, and/or a better understanding of soil P dynamics following liming, seems necessary to avoid this ABA-mediated response.

## Abbreviations:

ABA: abscisic acid
Ci: intercellular CO_2_ concentration
DW: dry weight
*g*_s_: stomatal conductance
Ѱ_leaf__,_ leaf water potential; Pn: photosynthesis
WT: wild type.
